# Functional cure of a chronic virus infection by shifting the virus - host equilibrium state

**DOI:** 10.3389/fimmu.2022.904342

**Published:** 2022-08-30

**Authors:** Gennady Bocharov, Dmitry Grebennikov, Paula Cebollada Rica, Eva Domenjo-Vila, Valentina Casella, Andreas Meyerhans

**Affiliations:** ^1^ Marchuk Institute of Numerical Mathematics, Russian Academy of Sciences, Moscow, Russia; ^2^ Moscow Center for Fundamental and Applied Mathematics at INM RAS, Moscow, Russia; ^3^ Institute for Computer Science and Mathematical Modelling, Sechenov First Moscow State Medical University, Moscow, Russia; ^4^ Infection Biology Laboratory, Department of Medicine and Life Sciences, Universitat Pompeu Fabra, Barcelona, Spain; ^5^ Catalan Institution for Research and Advanced Studies (ICREA), Barcelona, Spain

**Keywords:** Chronic virus infections, multi-stability, shifting equilibrium states, cure strategies, LCMV (lymphocytic choriomeningitis virus), HIV (human immunodeficiency virus)

## Abstract

The clinical handling of chronic virus infections remains a challenge. Here we describe recent progress in the understanding of virus - host interaction dynamics. Based on the systems biology concept of multi-stability and the prediction of multiplicative cooperativity between virus-specific cytotoxic T cells and neutralising antibodies, we argue for the requirements to engage multiple immune system components for functional cure strategies. Our arguments are derived from LCMV model system studies and are translated to HIV-1 infection.

## Introduction

Chronic virus infections like those with Human Immunodeficiency Viruses (HIV) and Hepatitis B (HBV) and C (HCV) viruses continue to threaten global health. A common feature of these infections is the persistence of virus antigen and the associated exhaustion of virus-specific T lymphocytes (1–5). Although the latter reduces immune-cell-mediated pathology, it is associated with a reduction of virus control that enables antigen persistence and has per se pathological consequences. For example, untreated HIV infection mediates CD4 T cell depletion, chronic immune activation, lymphoid tissue destruction and dysregulation of immune homeostasis (6, 7). Chronic hepatitis virus infections deteriorate liver functions and can lead to liver cirrhosis and hepatocellular carcinoma (8–10). Globally these 3 infections are carried by close to 400 million individuals and thus are a significant burden for public health care systems (11).

The existing approaches to treat chronic infections may be subdivided into 2 fundamentally different categories, (i) targeting of virus replication by antiviral drugs including interferons or therapeutic target cell modifications, and (ii) targeting virus-specific immune responses to improve host control by restoration of immune functions i.e. through therapeutic vaccination or immune checkpoint inhibitors that reinvigorate exhausted lymphocytes. While most of these options are still experimental, antiviral drugs are by far the most common therapeutic modality in use and very successful. For example, the current virus-specific anti-HCV drugs are highly potent and enable virus clearance in around 95% of infected individuals (12). Current antivirals against HIV can reduce virus loads to below detectable levels however fail to eliminate the latently infected cells (13, 14). As a consequence, treatment interruptions lead to rapid viral rebounds from viral reservoirs and the continuation of a high viral load infection state (15). To overcome the need for life-long antiviral HIV therapy with its side effects and the inherent financial burden for health care systems, numerous concepts for curing chronic infections have been developed and experimentally tested. These include “shock-and-kill” strategies that aim to purge the latent virus reservoir by latency reversal agents with subsequent killing of infected cells (16, 17), “block-and-lock” strategies that aim to permanently silence all HIV proviruses (18) and the potential “rinse-and-replace” strategy that predicts a “washing-out” of infected cells by uninfected naive and memory T cells *via* sequential waves of polyclonal T cell stimulation under combination antiretroviral therapy (19). While still being far from clinical practice, the combined delivery of broadly neutralising antibodies or CD8 T cell-inducing therapeutic vaccines with latency-reversal-agents (LRAs) including TLR7 agonists showed encouraging first results in experimental SIV/SHIV infections of rhesus monkeys (20, 21) and in humans (22).

**Figure 1:**
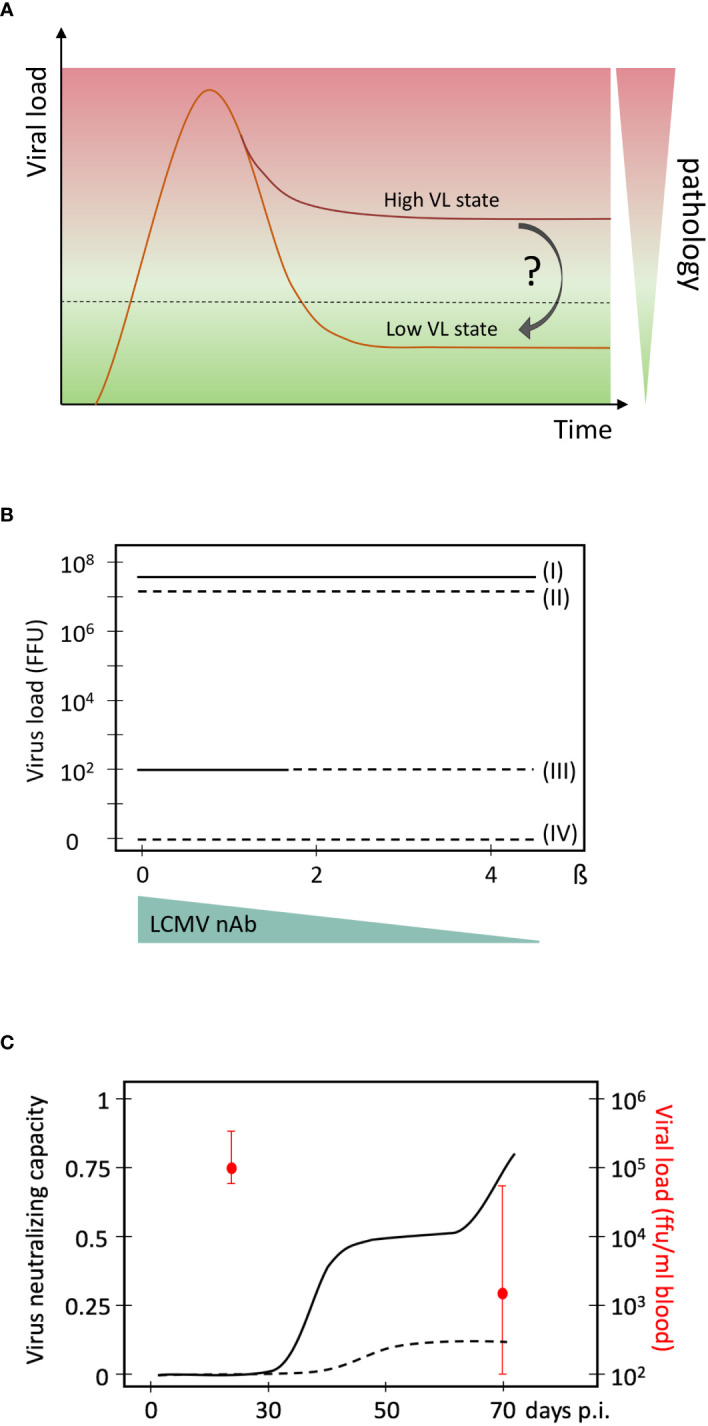
High and low virus load (VL) equilibrium states in persistent chronic virus infections. **(A)** Schematic presentation of a high and low virus load state, and the associated pathology. **(B)** Mathematical model predictions of multiple virus equilibrium states (I to IV) in LCMV infections of mice as a function of the net virus growth rate β. Data are from (28). The four virus equilibrium states differ in their values of the virus load from the highest (state I) to the lowest (state IV). Stability of an equilibrium state or steady state means that the system returns back after some perturbation. Only stable equilibrium states are biologically observable. State I is always stable while state III is stable for a certain range of β (solid lines). States II and IV are unstable (broken lines) and cannot be observed biologically. A possible reduction of β by neutralising antibodies is indicated below. It represents the natural occurrence of a late, specific neutralizing antibody response during chronic LCMV infection that reduces the net virus growth rate represented by β. **(C)** Evolution of virus neutralising capacity of mouse sera during an experimental LCMV infection of mice. Experimental data are from (29) and converted into this presentation. Medians of viral loads at days 30 and 70 and their interquartile ranges are indicated in red. Solid line, sera from chronic infection; broken line, sera from acute infection.

Cure strategies for chronic virus infections can be divided into sterilising and functional cure strategies. While the former attempts to completely eliminate the virus from the host e.g. by HIV-resistant hematopoietic stem cell transplantation (23, 24) or provirus deletion approaches (25–27), the latter solely aims to shift the dynamic virus - host immune system balance into a state in which the virus is sufficiently controlled without causing pathology (**Figure 1A**). Given that HIV elimination was only successful in possibly 4 cases worldwide with a procedure that has a high mortality rate (30), functional cure approaches appear more feasible. However, is there any evidence that a shift from a high-virus-load to a low-virus-load equilibrium is possible? And what would the requirements be for such a shift? Here, we discuss the evidence that indeed such a shift should be possible and define the conditions under which it may occur.

## LCMV model system-based analyses

Many features of virus - immune system interactions within the lymphocytic choriomeningitis virus (LCMV) mice model system resemble those of human chronic HIV and hepatitis virus infections (31, 32). In the early stages, LCMV infections are mainly controlled by CTLs. Infection with a low dose of e.g. LCMV-Docile or a high dose of LCMV-Arm leads to the clearance of virus below the detection level and formation of immune memory (33). In contrast, infection with high doses of LCMV-Docile or LCMV-Cl13 results in chronic viral persistence at high levels and exhaustion of antigen-specific cytotoxic CD8 T cells (CTLs) (1, 34). Nonetheless, they differ in their long-term kinetics of infections i.e. clearance of LCMV Clone 13 by late neutralizing antibodies versus persistence of LCMV Docile (1).

To explore the necessary conditions for the co-existence of virus-host equilibria that differ in viral loads as well as the possibilities for transferring a high-viral load state to a more favourable equilibrium, one can utilise the analytical power of existing mathematical models that have been calibrated using experimental data. Our previously developed mathematical model of LCMV infection considers the population dynamics of viruses, precursor and effector CTLs (35), and utilises LCMV data assimilation procedures and bifurcation analysis (36). The results suggested that the reduction in the net viral growth rate β is a necessary condition for a stable low level LCMV infection state within an immunocompetent host (**Figure 1B**). Specifically, the existence of replication competent LCMV below the detection limit of about 100 FFU per spleen in immune mice requires a more than 2-times reduction of the exponential virus growth rate of the acute infection phase. Given that LCMV-specific neutralising antibodies (nAbs) can block free virus particles and thus reduce the net virus growth rate, it was hypothesised that such antibodies could be decisive for virus control. And indeed, subsequent experimental work by Greczmiel *et al.* demonstrated that it is the late appearance of nAbs that finally controls a chronic LCMV infection to below detectable levels (37) (**Figure 1C**).

**Figure 2:**
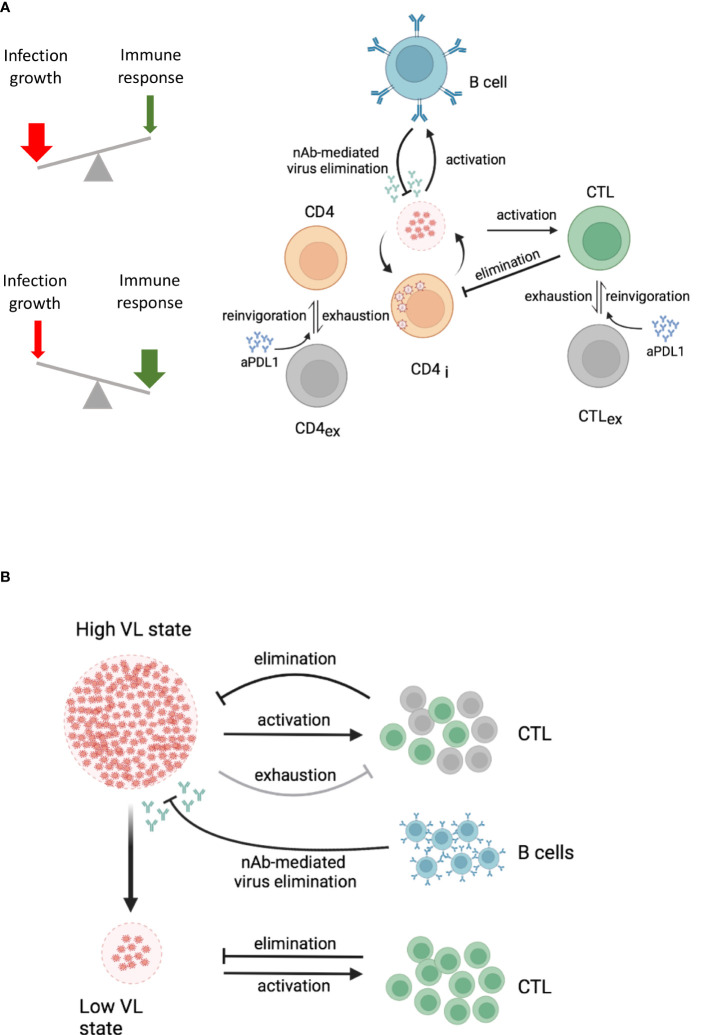
Dynamic views of virus-host interactions. Lines with an arrow or T end represent expansion or reduction/suppression of the corresponding virus or cell populations, respectively. The specific processes by which this occurs are specified above the lines. **(A)** Conceptual dynamic view of a high and a low virus load state within an infected host. The underlying processes are indicated. A high VL state drives CTL exhaustion (grey cells) and reduces the population of effector CTL (green cells). B cells (blue cells) produce antibodies that eliminate infectious viruses. Both, effector CTL and antibodies from B cells, depending on their strength, contribute to the control of the virus load. **(B)** Cooperative engagement of individual immune components for functional cure strategies in HIV-1 infection. The combination of immune checkpoint inhibitors with CTL-based immunotherapy and neutralising antibody responses is indicated. aPDL1, anti-PDL1 antibodies; CTL, cytotoxic T cells; CTLex, exhausted CTL; CD4i, infected CD4 T cells; VL, virus load. The figure was created with BioRender.com.

A conceptual dynamic view of the above observations is summarized in **Figure 2A** which considers the outcome of virus-host interactions as a ‘numbers game’ between the rate of infection growth and the activation of the immune system (38). If the virus outcompetes the CTL response, a high virus load state is established that is characterised by T cell exhaustion and maintained through the interaction of inhibitory receptors on T cells with their ligands on antigen-presenting cells (APCs) (5). However, this harmful equilibrium can be shifted in favour of the host by inducing a virus-specific neutralising antibody response or by providing antibodies as a therapeutic intervention (37, 39). Since the cooperativity of remaining CTLs and the newly induced antibody response can be considered as multiplicative rather than just additive, the demand for both specific immune response components is less stringent in absolute numbers (40).

## Extrapolation to HIV infections

To translate these results and considerations from the LCMV mouse model system to human infections like HIV infections and functional cure strategies, two fundamental questions arise: Is there evidence for different dynamic steady states in HIV infection? How can one reduce viral growth rate β and restore functional immune control in a therapeutic setting? Answer to the first question is yes. There are several different dynamic states that can be defined by viral loads and disease progression rates (41). These are related to the virus set point, the dynamic equilibrium state at which the virus settles after the primary infection phase (42–44). For example, from the infected individuals in which the virus load settles to above 36,000 virions/ml blood, more than 62% will develop AIDS within 5 years. In contrast, only 8% of individuals with a virus set point below 4,500 virions/ml blood will develop AIDS in this time frame. Thus, at least in HIV-1 infection, virus loads are directly linked to pathology and the low virus load stage, as observed in so-called “elite controllers” (EC) (45, 46), may be regarded as a non-pathogenic virus infection state that should be the target for functional cure strategies (**Figure 1A**). Indeed, elite controllers are exceptional HIV-infected individuals that control virus replication without the requirement of antiretroviral therapy. Based on studies during their chronic steady state, many potential immunological and virological factors have been linked to this. It includes virus-specific CTLs and CD4 T cells, innate immune responses like NK cells and plasmacytoid dendritic cells as well as virus attenuation and provirus integration into repressed chromatin sites (47–49). Since the EC state is associated with certain HLA types, and the antiviral functionality of CTLs from EC against HIV-infected autologous CD4 T cells is superior to those of HIV non-controllers (50), HIV-specific CTL seem to be a prime component in achieving virus control. The role of virus-specific neutralizing antibody responses in EC is less clear as high titres of these are often lacking in respective individuals (47).

The answer to the second question is much more demanding because one needs to consider the state of the whole immune system and virus population at the time the functional cure strategy is to be initiated. In the chronic infection state in which T cells are exhausted (5) and the virus is a complex quasispecies population (51, 52) and partially hiding within latently infected cells (53, 54), the simple reduction of the virus growth rate by antiviral drugs is not sufficient to self-maintain a stable low virus load state. Likewise, it is not sufficient to invigorate exhausted T and B cells by checkpoint inhibitors in the presence of antiviral drugs because only a fraction of HIV disease phenotypes would benefit. This was demonstrated in our modelling study of anti-PD-L1 blockage in HIV-1 infection (55). In this we showed that a favourable effect in terms of viral load reduction and restoration of functional T cells strongly depends on the antibody-mediated elimination rate of infectious virus in a threshold dependent manner. Furthermore, taking into account spatial aspects of HIV-1 infection spreading within lymphatic tissue and CTL motility, we estimated that the minimum frequency of HIV-specific effector CTLs should be above 5% to ensure localisation and elimination of an infected cell within a virus life cycle time (56). Recent vaccine studies against simian–human immunodeficiency virus (SHIV) infection in macaques demonstrated that the threshold requirements for virus infection control were much lower when neutralizing antibodies and CTLs were induced (57) suggesting multiplicative cooperation between both arms of the adaptive immune system (40). Taken together and considering the multiplicative cooperativity between cellular and humoral responses, it would appear that only a multi-modal empowerment of antiviral immunity could enable a permanent shift from a high virus load to a low virus load state in HIV-1 infection. This would require invigoration of exhausted T cells by checkpoint inhibitors in the presence of antiviral drugs (58) and induction of novel CTLs together with neutralising antibodies that cover a broad spectrum of viral epitopes (**Figure 2B**).

## Discussion

Here we summarise the evidence for multiple stable virus load states in persistent chronic virus infections and provide a perspective for a functional cure.

Multi-stability is a relevant property of complex biological systems with normal feedback regulation (59) to which virus infections belong to. It provides the possibility of switching between different virus load states. Computational models are helpful in this context as they can identify the required parameter values for the multiple steady states (28). For example, a 2-times reduction of the net virus growth rate β is the necessary condition for the existence of the low virus load state in persistent LCMV infection (**Figure 1B**). Once this condition is identified, the challenge becomes to define the required manipulations for shifting the whole system to that favourable state. Again, computational approaches can provide useful insight. Amongst them is the recently developed optimal disturbance approach which can predict multi-modal impacts (combination therapies) with maximal effects on the immune system (60). When applied to LCMV infection, the results demonstrated that a systems shift is possible and requires a combination of different initial state perturbations like virus load and functional T cell state. While this will translate to a combination therapy e.g. of checkpoint inhibitors and neutralising antibodies, the respective quantities and time lines are not yet determined and await experimental elaboration. Nonetheless, these mathematically-driven experimental LCMV system-based studies provide a proof-of-concept for a possible system shift to a favourable virus-host interaction dynamics. Respective analyses of multi-stability and optimal disturbances for HIV-1 or other persistent chronic infections in humans are still lacking and clearly represent a challenge and direction for further interdisciplinary research. The recent progress of immunotherapies to induce and boost antiviral immunity are encouraging but also highlight the need to cooperatively engage individual immune system components that may eventually allow moving from a drug-based virus containment to a long-term immune system-based functional cure.

In summary, the currently explored strategies for functionally curing an HIV infection are “shock-and-kill”, “block-and-lock” and “rinse-and-replace”. None of them considers and explores the concept of multiplicative cooperativity between individual immune system components that is proposed here. A proof of concept in a clinical setting is eagerly waited for.

## Data availability statement

The original contributions presented in the study are included in the article/supplementary material. Further inquiries can be directed to the corresponding authors.

## Author contributions

All authors listed have made a substantial, direct and intellectual contribution to the work, and approved it for publication. All authors contributed to the article and approved the submitted version.

## Funding

The authors are supported by the Russian Science Foundation (RSF grant no. 18-11-00171), the Russian Foundation of Basic Research (RFBR grant no. 20-01-00352), “la Caixa” Foundation under the project code HR17-00199, the Spanish Ministry of Science and Innovation grant no. PID2019-106323RB-I00 AEI//10.13039/501100011033, and “Unidad de Excelencia María de Maeztu”, funded by the MCIN and the AEI (DOI: 10.13039/501100011033) Ref: CEX2018-000792-M.

## Conflict of interest

The authors declare that the research was conducted in the absence of any commercial or financial relationships that could be construed as a potential conflict of interest.

## Publisher’s note

All claims expressed in this article are solely those of the authors and do not necessarily represent those of their affiliated organizations, or those of the publisher, the editors and the reviewers. Any product that may be evaluated in this article, or claim that may be made by its manufacturer, is not guaranteed or endorsed by the publisher.
